# Performance Evaluation of GNSS Position Augmentation Methods for Autonomous Vehicles in Urban Environments

**DOI:** 10.3390/s22218419

**Published:** 2022-11-02

**Authors:** Harihara Bharathy Swaminathan, Aron Sommer, Andreas Becker, Martin Atzmueller

**Affiliations:** 1 Semantic Information Systems Group, Osnabrück University, 49090 Osnabrück, Germany; 2 Aptiv Services Deutschland GmbH, 42119 Wuppertal, Germany; 3 Faculty of Information Technology, Dortmund University of Applied Science and Arts, 44139 Dortmund, Germany; 4 German Research Center for Artificial Intelligence (DKFI), 49090 Osnabrueck, Germany

**Keywords:** GNSS, GPS, positioning, position augmentation, Applanix POS LV system, RTK, Trimble RTX, autonomous cars

## Abstract

Global Navigation Satellite Systems provide autonomous vehicles with precise position information through the process of position augmentation. This paper presents a series of performance tests aimed to compare the position accuracy of augmentation techniques such as classical Differential Global Navigation Satellite System, Real-time Kinematic and Real-time eXtended. The aim is to understand the limitations and choose the best position augmentation technique in order to obtain accurate, trustworthy position estimates of a vehicle in urban environments. The tests are performed in and around the German cities of Wuppertal and Duesseldorf, using a vehicle fitted with the navigation system POS-LV 220, developed by Applanix Corporation. In order to evaluate the real-time performance of position augmentation techniques in a highly challenging environment, a total of four test regions are selected. The four test regions are characterized mainly by uneven terrain with tall buildings around the University of Wuppertal, flat terrain with roads of varying width in the city centre of Wuppertal and Duesseldorf and flat terrain in a tunnel section located in the city of Wuppertal. The performances of the different position augmentation are compared using a Root Mean Square (RMS) error estimate obtained as an output from the Applanix system. Furthermore, a High-Definition map of the environment is used for the purpose of model validation, which justifies the use of RMS error estimate as an evaluation metric for the performance analysis tests. According to the performance tests carried out as per the conditions specified in this paper, the Real-time eXtended (RTX) position augmentation method enables to obtain a more robust position information of the vehicle than Real-time Kinematic (RTK) method, with a typical accuracy of a few centimeter in an urban environment.

## 1. Introduction

Precise navigation is one of the most important requirements of a fully autonomous car. The car should possess the knowledge of its own position and orientation, in order to navigate precisely within and through an environment [1]. Current road cars use the Global Positioning System (GPS) [2,3,4], an example of the Global Navigation Satellite System (GNSS) which aids the car for estimating its own position. In a navigation system involving standalone GPS, a raw position information signal originates from a geo-stationary satellite and reaches a receiver located on the earth surface. During this phase of travel, pseudo-range errors occur due to the solar radiation in the ionosphere and weather conditions such as clouds, rain and lightning in the troposphere [5]. Even though GPS is used by road cars to estimate their position, its accuracy is only up to a few meters under open sky conditions and the signal is heavily influenced by the aforementioned factors resulting in inaccurate position estimates [6]. As per the Federal Aviation Administration GPS Performance Analysis Report [7], the accuracy of standard GPS is within 3 m, which is insufficient for a fully autonomous car which requires a more precise localization. In contrast to road cars, a fully autonomous car requires an position accuracy of a few centimeter with very high reliability and precision, in order to maintain lane-level accurate localization. Therefore, an improved navigation system is used to estimate the position through augmentation of the raw signal with additional information. One method for improving attributes of a navigation system such as accuracy, reliability and availability, through the integration of external information into the position calculation process is known as Global Navigation Satellite System (GNSS) position augmentation. Some of the widely known methods include Differential Global Navigation Satellite System (DGNSS) [8], Real-time Kinematic (RTK) [9] and Real-time eXtended (RTX) [10].

In this paper, we conduct a series of performance tests aimed to compare the position augmentation accuracy of classical DGNSS, RTK and RTX in the cities such as Wuppertal and Duesseldorf located in Germany. As part of the comparative evaluation, the position augmentation methods are compared using Root Mean Square (RMS) values obtained from the navigation system itself. Our results indicate, that the positional updates obtained using Real-time eXtended process are more robust than Real-time Kinematic with an accuracy of a few centimeter in an urban environment.

Our main contributions are summarized as follows:

1. The experiments in this paper were carried out with a reference base station located few kilometers away as compared to experiments with the reference station located within a kilometer. In order to obtain results indicative of real-time challenges posed to GNSS signal availability, test regions consisting of both flat and uneven terrain posing various challenges to signal availability were selected for analysis.
2. The performance analysis presented in this paper helps in identifying a precise position augmentation method for the purpose of localizing autonomous vehicles in dynamic environments particular to German cities such as Wuppertal and Duesseldorf, characterized by regions consisting of buildings, tunnels and underpasses.

## 2. Related Work

Until now, Scherzinger [11] presented the performance of Position and Orientation System for Land Vehicles (POS LV) with Inertially Aided Real-time Kinematic position augmentation system (IARTK) through one of the tests in downtown Tokyo to study the behavior of Applanix POS LV system during partial and total GPS Outages. In their paper, a tightly coupled inertial-GPS integration was introduced for obtaining inertially aided RTK position updates. Through this way of implementation, the navigation solution was able to provide position updates even during phases when fewer than four satellites were visible. Furthermore, the tightly coupled inertial-GPS integration enabled position updates with an accuracy of several meters during partial and total GPS outage situations.

Zywiel [12] described the performance results of a test where a van equipped with an Applanix POS LV aided inertial navigation system was driven through a suburban area located north of Toronto, Canada. Frank and Louis [13] presented the results of a test carried out in downtown Toronto to determine the operational capabilities of the Applanix POS LV 200 system. In their paper, the position accuracy during the test is presented using Root Mean Square (RMS) values obtained directly as output from the POS LV 200, which we have also followed for performance analysis presented in the later sections of this paper. The aforementioned works established the ability of aided inertial systems to provide a continuous, accurate position information in urban environments.

Erol et al. [14] presented a performance analysis of real-time position coordinates obtained through the commercial service, the Trimble CenterPoint RTX in a maritime environment. In an experiment carried out on a lake dam in Turkey, the Trimble CenterPoint RTX was capable of achieving centimeter level accuracy. This study implied that a real-time position service such as the CenterPoint RTX is a powerful tool for obatining highly accurate position even for dynamic applications. Ng et al. [15] evaluated the performance of RTK in land area that is obstructed by buildings and in uneven landscape. It was evident that the position accuracy of RTK was affected due to factors such as uneven landscape and blockage of signal from the base station by buildings. Overall, to the best of authors’ knowledge, an analysis comparing the accuracy of various GNSS position augmentation methods such as the DGNSS, RTK and RTX with focus on a dynamic urban environment has not yet been performed.

The rest of this paper is organized as follows. Section 3 gives an overview about Global Navigation Satellite System and Position augmentation methods such as RTK and RTX. Then, Section 4 introduces main components of the vehicle used for data collection and evaluation metrics. After that, Section 5 discusses the results of performance analysis. Finally, Section 6 concludes the paper with a summary and outlines interesting directions for future research.

## 3. Background: An Overview on the Global Navigation Satellite System

Global Navigation Satellite System is a term that is broadly used to refer all satellite-based systems comprising a constellation of satellites orbiting over the earth’s surface and continuously transmitting signals enabling users to determine their position on earth. One universally well-known system of GNSS is the Global Positioning System, developed by the US Department of Defence for public use. Other systems of GNSS currently include the Russian Global Navigation Satellite System (GLONASS), the European satellite system (Galileo) and China’s Beidou. Furthermore, regional satellite navigation systems such as Quasi-Zenith Satellite System (QZSS) operated by Japan and Indian Regional Navigation Satellite System (IRNSS) are also part of GNSS [16]. A GNSS-compatible hardware equipment is capable of using navigational satellites from networks beyond the GPS system for increased position accuracy and reliability.

Each GNSS, like the GPS, comprises of three major components such as the space segment, control segment and user segment. The space segment consists of GNSS satellites arranged in a constellation, orbiting about 20,000 Km above the earth’s surface. Each satellite transmits two carrier waves, referred to as L1 and L2 with information related to time, the satellite’s orbital position and status. The control segment is made a ground-based network of master control stations, data uploading stations and monitor stations. The GPS comprises of two master control stations, four data uploading stations and 16 monitor stations located throughout the world. In a GNSS system, the master control station adjusts the satellite position information and onboard high-precision clocks when necessary to maintain accuracy. The monitor stations installed over a broad geographical area monitor the satellite signals and status and relay this information to the master control station. The master control station analyses the signals and then transmits position and time corrections to the satellites through data uploading stations. The user segment consists of equipment that processes the received signals from the GNSS satellites and uses them to derive location and time information. The equipment used in the user segment could be a smartphone or an improved receiver for survey and mapping purposes by an autonomous vehicle. Each of this equipment has one or two antennas to receive either one or both carrier signals sent by a satellite depending upon the accuracy requirements and a receiver to process the radio signals, calculate position and time [17]. A standalone GNSS service such as the GPS is accurate only within a few meters and does not satisfy the position requirements of a fully autonomous vehicle as it is susceptible to a wide range of errors, namely the time delay introduced by the layers of the atmosphere, the ionosphere and troposphere, effects such as multipath due to reflections from nearby objects. In addition, errors introduced on satellite clocks and orbit information also result in inaccurate position. Therefore, techniques and equipment have been developed to improve the accuracy and availability of GNSS position and time information. In the remaining part of this section, techniques introduced to improve the accuracy and availability of GNSS are explained.

**Figure 1:**
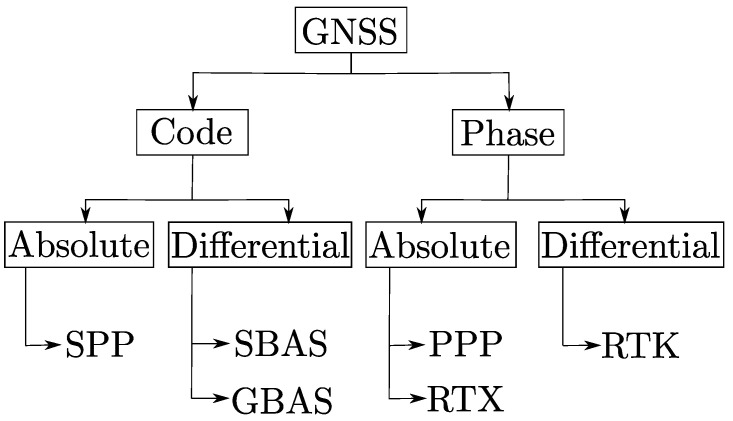
Classification of GNSS systems.

GNSS systems can be broadly classified into code-based and carrier phase-based measurement according to the technique used by the receiver to calculate the travel time of the satellite signal. A receiver determines the time by comparing a pseudo random code generated by itself, with an identical code in the signal. In case of GPS, the pseudo random code is referred as the coarse acquisition C/A code which is freely available to public users. This process of matching codes to estimate the travel time of a signal is termed as code-based measurement and is highly prone to errors. On the other hand, the phase-based measurement technique is performed by the receiver estimating phase differences between L1, L2 carrier waves to achieve millimeter level precision. Furthermore, GNSS techniques are also classified into absolute and differential depending upon the nature in which the receiver determines position. In absolute GNSS, a modeling of random error sources are carried out to calculate the position. On the other hand, a differential GNSS involves the use of correction data from a base station whose position is known to a high degree of accuracy for the calculation of position. The overall classification of GNSS systems is shown in Figure 1.

A Space Based Augmentation System (SBAS) [18,19,20] uses a network of reference base stations on the ground to obtain positional error data for a particular area. Subsequently, this information is sent up to a geo-stationary satellite before being broadcasted back down to all receivers in a particular area. In comparison to SBAS, a Ground Based Augmentation System (GBAS) [21,22], is a complete on-ground facility to provide corrections. Both of these systems are accurate up to 1 m. Real-time Kinematic (RTK) is a technique widely used to receive precise position information using corrections that are transmitted via some sort of a data communication (e.g., 3G/LTE connection) from a single base or multiple reference base stations with an accuracy of less than 2 cm. The main drawback of the single base RTK technique is the necessity of a reference station located close to user. For a large area, the RTK is extended from a single base to a multi-base technique. The major success of RTK is attributed to the development of Network RTK which resulted in a tremendous reduction of the investment costs necessary to start an RTK service, since the number of reference stations can be reduced [23]. In case of RTK, the availability of corrections is dependent on the prevailing environmental conditions. For example, a rugged terrain condition could possibly interrupt the correction signal, even if the distance between the base station and the receiver is short [14]. Some providers of RTK services are SAPOS [24] with over 270 stations in Germany and Trimble VRS Now [25] with 205 stations throughout Europe. In the Precise Point Positioning (PPP) method, the data collected with a single GNSS receiver is used with precise satellite orbit and clock information from data analysis centres such as the International GNSS Service (IGS) to achieve centimeter level position accuracy. However, the requirement of relatively long convergence time to achieve the cm-level precision is one of the main drawbacks of this method, thereby limiting the use of PPP on real-time applications [14]. The Trimble CenterPoint Real-time eXtended (RTX) belonging to Real-time PPP (RT-PPP) method is a type of GNSS correction service that provide position accuracy of a few centimeter through an L-band satellite communication or Internet. In contrast to RTK, RTX does not require the local reference station as Satellite orbits, satellite clock offsets and atmospheric errors are estimated in real-time for any place on the globe using innovative and advanced data processing algorithms by analysis centres.

## 4. Method

In this section, the vehicle setup and the evaluation metric used for the comparison of GNSS position augmentation methods is described.

### 4.1. Vehicle Setup

**Figure 2:**
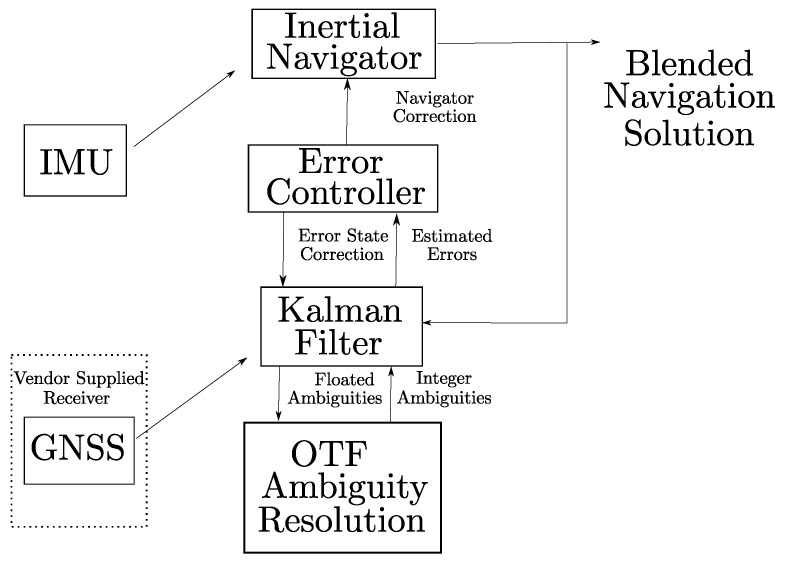
Block diagram of components within an integrated inertial system such as the Applanix POS LV-220 [13].

The Applanix POS LV-220 system, developed and manufactured by Applanix: A Trimble Company based in Richmond Hill, Ontario, Canada, is mounted onto the test vehicle consisting mainly of an inertial measurement unit (IMU), a GNSS signal receiver, an Inertial navigator component and a error controller component consisting of a Kalman filter and an Error controller [26]. A block diagram consisting of all the components within an integrated inertial system such as the Applanix POS LV-220 is shown in Figure 2. In the Applanix POS LV-220, the GNSS signal receiver is configured to receive position updates such as RTK and RTX through a cellular network. Due to the fact that a combination of GNSS techniques to obtain position and IMU is used to generate accurate, continuous position and orientation information, the Applanix POS LV-220 is termed as an integrated inertial system. The IMU comprises of accelerometers and gyroscopes to measure the 3D accelerations and orientations. Even though a complete navigation solution can be provided by using IMU without any external information, the position and orientation errors grow unbounded over time in such a free-inertial system due to bias errors [27]. This drawback of a free-inertial system is overcome by using GNSS position updates to aid the IMU and thereby decrease the position and orientation errors. Therefore, the Applanix POS LV-220 system consists of a GNSS receiver to receive positional updates through GNSS position augmentation methods mentioned in the earlier section. The inertial navigator component receives accelerations and angular rates from the IMU’s accelerometers and gyros to compute the IMU position, velocity and three-axis attitude such as roll, pitch and yaw. Since the computed components of the inertial navigator are prone to error such as initialization and inertial sensor errors, an Error controller component is used for controlling these errors by adjusting the Inertial navigator integration process and calibrating the gyro and accelerometer. The error regulation component comprises the Kalman filter and the closed-loop error controller. The Kalman filter maintains a multivariable linear stochastic model of the inertial navigator errors as mentioned in [28]. The Kalman filter is a recursive least squares estimator that computes optimal estimates of errors from measurement of position and velocity computed by the inertial navigator differenced with corresponding position and velocity from the embedded GNSS receiver. By estimating the accelerometer and gyro biases, the Kalman filter effectively calibrates the inertial sensors dynamically during normal operation. The error controller computes resets to the inertial navigator integration processes based on the Kalman filter estimates, and applies these so as to cause the estimated errors to be nulled. Due to this error control loop, the initial alignment of the inertial navigator and subsequent continuous error regulation in all navigation quantities are achieved. As the errors in the inertial navigation component are continuously and optimally controlled by the error regulation component, this integration is termed as “tightly-coupled” [29]. As an output from the Applanix POS LV-220 system, the position information consisting of latitude, longitude and height, the heading, roll and pitch of the vehicle is available at all times.

### 4.2. Evaluation Metric

In the Applanix POS LV-220 system, the performance metrics are expressed in meters with North RMS error, East RMS error and Down RMS error corresponding to Northing, Easting and Altitude coordinates, respectively. All these RMS errors are scaled estimates provided by the navigation system based on prevailing conditions such as the number of satellites visible, the quality of signal, etc. influencing the accuracy of position coordinates. In this research, a single RMS value calculated by averaging the North RMS error and East RMS error is used to denote positional accuracy. Therefore, an RMS value close to zero is indicative of precise position than a much higher value.

## 5. Results

**Figure 3:**
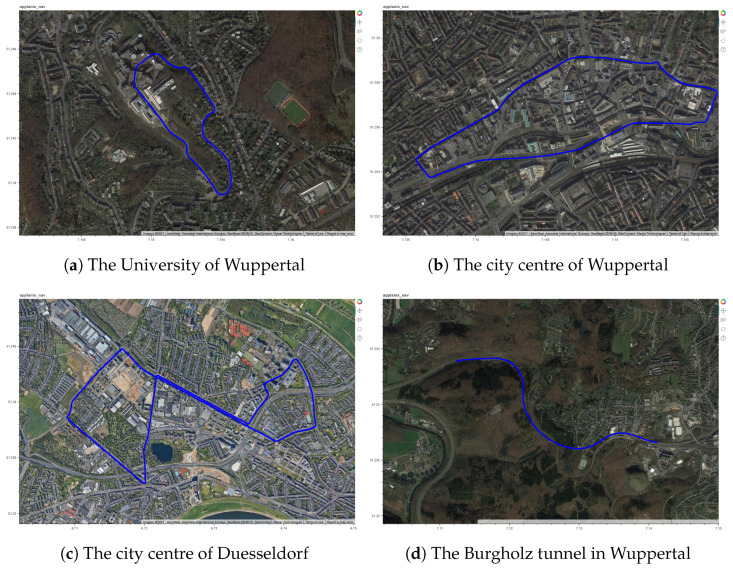
The selected test regions for performance analysis.

A total of four different test regions were selected based on varying levels of traffic congestion, challenges to signal availability due to the presence of tall buildings and trees in the region. The path driven by the vehicle in these test regions are shown in Figure 3. The test region shown in Figure 3a is located around the University of Wuppertal and is characterized by few tall buildings, an underpass and also a residential area. A single trip around this test region covers a distance of 1.89 km with an elevation gain/loss of ±50 m and an average slope of 4.4%. The test region 2 shown in Figure 3b is located in the city centre of Wuppertal. This region is characterized with narrow road sections and highly dense traffic. A lap around the test region covers a distance of 3.43 km with an elevation gain/loss of ±63 m and an average slope of 3.1%. The test region 3 shown in Figure 3c is located in the city centre of Duesseldorf. This region comprises of streets with medium and highly congested traffic. A single lap around this test region covers a distance of 7.88 km with an elevation gain/loss of ±86 m and an average slope of 1.6%. Finally, the test region 4 shown in the Figure 3d comprises of the Burgholz tunnel which is 1865 m long and facilitates a direct and continuous expressway connection between the German motorways (Autobahn) A1, A46.

**Figure 4:**
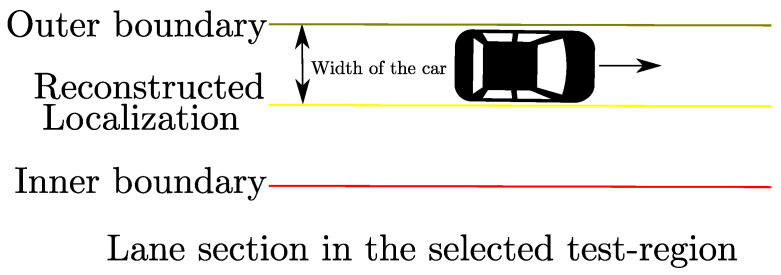
Approximation of vehicle position (in Yellow) in between lanes using lane dimensions of the selected test-region obtained from HD map and vehicle width dimension.

It is essential to carry out model validation to ensure whether large RMS values are indicative of position degradation as these values are estimated by the Applanix solution, being used for performance evaluation of position augmentation methods in this paper. For this purpose, a small section of the test-region is selected in such a way that the test vehicle can be localized approximately. Due to the absence of ground truth, it was ensured that the vehicle was driven close to the outer lane boundary in this test region as shown in Figure 4. For the purpose of localizing the vehicle laterally between the inner boundary and outer boundary of the lane section, the width of test vehicle is considered. For this approximation, the lane dimensions were obtained from an HD map with position accuracy of less than 2 cm, provided by 3D Mapping Solutions GmbH, Holzkirchen, Germany [30].

**Figure 5:**
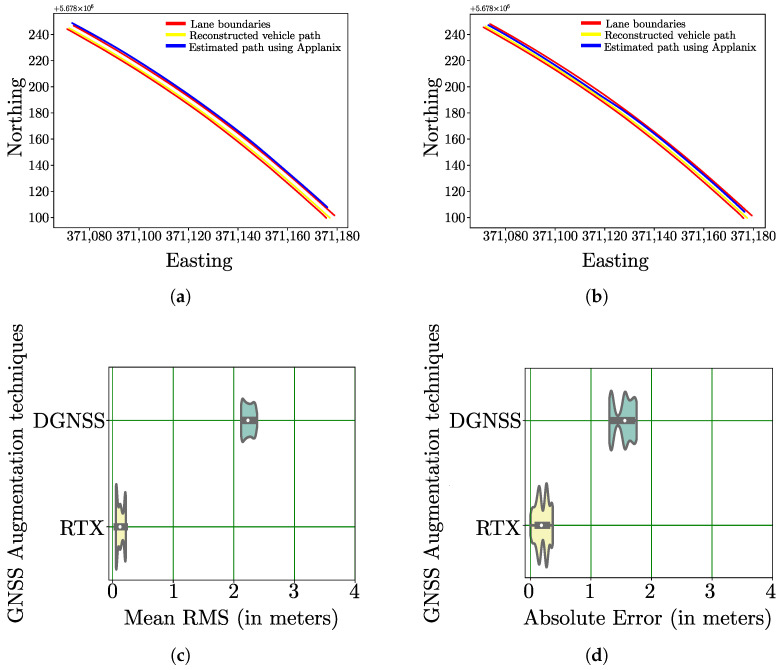
Model validation to establish relationship between high RMS values and position degradation. (**a**) Reconstructed Vs Estimated path using DGNSS position updates of the test-vehicle. (**b**) Reconstructed Vs Estimated path using RTX position updates of the test-vehicle. (**c**) Mean RMS as derived from Applanix POS LV-220 system. (**d**) Absolute Error as calculated by using reconstruction.

In Figure 5a, the reconstructed path of the test vehicle (in yellow), the lane dimensions obtained from the HD map (in Red) and the estimated path of the test-vehicle using DGNSS position updates (in Blue) are plotted. Similarly, Figure 5b comprises of the reconstructed path of the test vehicle, the lane dimensions obtained from the HD map and the estimated path of the test-vehicle using RTX position updates. In order to determine the error at an estimated position on the test-region, a metric termed as Absolute error is used. The Absolute error for every position (*p*) on the test-region estimated by the Applanix solution can be determined by finding the nearest point (*q*) on the approximated position of the test vehicle based on the euclidean distance. Each position (*p*,*q*) in the test-region is assigned a Universal Transverse Mercator (UTM) coordinate as the UTM is a system for assigning coordinates to locations on the surface of the earth. According to the UTM system, each position on the surface of earth is assigned an Easting (m), a Northing (m), a Down (m) and a UTM zone value. The Absolute Error is the mean of two individual position error components: the Easting Error and a Northing Error. For every position estimate obtained from the Applanix solution, the individual error components and the overall Absolute Error can be determined in the following way:

(1) $$Error_{easting}=\left|q_{Easting}-p_{Easting}\right|\;$$

(2) $$Error_{northing}=\left|q_{Northing}-p_{Northing}\right|\;$$

(3) $$Absolute\;Error=\frac{\left(Error_{easting}+Error_{northing}\right)}{2}\;$$

In Figure 5c,d, violin plots showing the distribution of Mean RMS values estimated from the Applanix solution and the calculated Absolute Error for the selected region are provided. In a violin plot, a kernel density estimation is used to represent the distribution shape of data on either sides of the thin grey line. In particular, the wider sections are used to represent a higher probability that members of the population will take on the given value and the skinnier sections represent a lower probability. The thick grey bar in each violin represents the interquartile range and thin grey line indicates rest of point distribution excluding the outliers. Finally, the white dot represents the median value. From the violin plots, it can be observed that high Mean RMS values are indicative of high position error as exhibited by DGNSS and low Mean RMS values are indicative of better positioning performance as exhibited by RTX. As a result of this model validation, the mean RMS values estimated by the Applanix solution are henceforth used as an evaluation metric for the performance analysis across all the test-regions.

**Figure 6:**
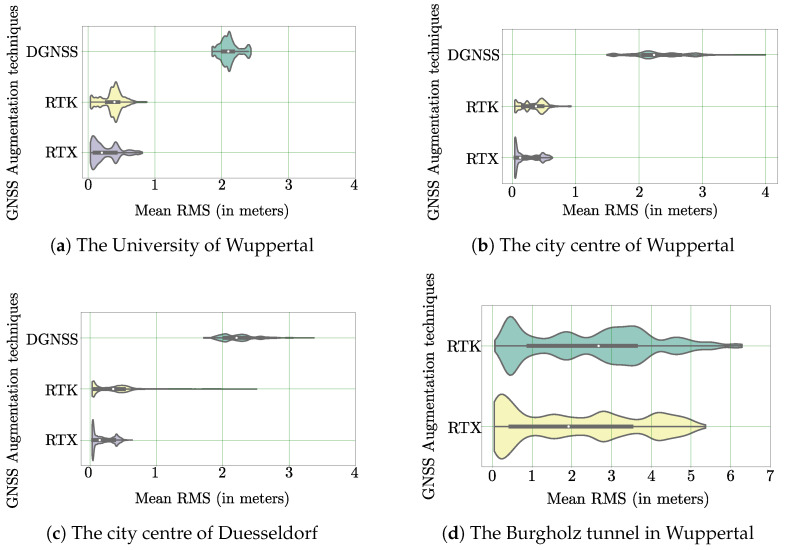
Horizontal violin plots showing the distribution of RMS values across various position augmentation techniques for each test region. The white dot represents the median RMS error value, the thick grey bar in the center represents the interquartile range and the thin grey line represents the rest of the distribution.

In order to compare the positional accuracy across various position augmentation techniques such as DGNSS, RTK and RTX in the selected test regions, horizontal violin plots showing the distribution of RMS values are used. Figure 6 consists of these horizontal violin plots that depict the positional accuracy of the augmentation techniques across the selected test regions. In particular, Figure 6a depicts the test region around the University of Wuppertal, Figure 6b depicts the test region located in the city centre of Wuppertal, Figure 6c depicts the test region located in the city centre of Duesseldorf and Figure 6d depicts the test region consisting of the Burgholz tunnel in Wuppertal.

It is observed from the violin plots of test regions shown in Figure 6a–c that the RMS values of DGNSS-based position augmentation are far higher than the values estimated with techniques such as RTK or RTX. The fluctuation of positional accuracy with DGNSS, particularly in a complete urban environment such as the city centre of Duesseldorf, Wuppertal is observed with the length of thin grey line in Figure 6b,c. The aforementioned behaviour of DGNSS is due to the use of an outdated protocol. On the other hand, RTK and RTX uses sophisticated protocols such as the CMR, CMR+ or RTCM 3 to perform error calculation for correcting the ionospheric delays and satellite clock errors. Furthermore, the deterioration of RTX and RTK correction signals inside the Burgholz tunnel resulted in the Applanix system switching to a less accurate position augmentation modes. This can be observed in Figure 6d, where the range of RMS values for the performance of RTK and RTX is higher than the values observed for other test regions. The range of RMS values for the performance of RTK and RTX is higher in the test region involving the Burgholz tunnel because the tunnel is preventing the GNSS receiver from receiving any correction or positioning signal termed as GNSS outage. As a result of this, the errors in the IMU accumulate over time.

A detailed percentage ratio of data samples acquired during the performance test carried out in the test region consisting of the Burgholz tunnel is shown in the Table 1 and Table 2. In particular, Table 1 shows the percentage distribution of data samples acquired during the laps with RTK position augmentation. During the course of four laps driven through the Burgholz tunnel with RTK position augmentation, the majority of samples were acquired from methods with accuracy close to 75 m and 1 m.

In a similar way, Table 2 shows the percentage distribution of data samples acquired during the laps with RTX position augmentation. The percentage distribution of data samples observed during these laps is similar to the behavior observed during RTK position augmentation with the majority of samples acquired from methods with accuracy close to 75 m and 1 m.

**Figure 7:**
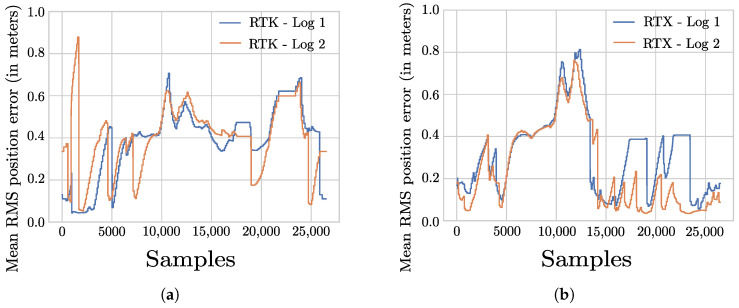
RMS values estimated by the Applanix POS LV-220 system during the course of laps around the University of Wuppertal. (**a**) Estimated RMS values during RTK position updates. (**b**) Estimated RMS values during RTX position updates.

The expected accuracy of position augmentation methods given in the column names of Table 1 and Table 2 were provided by Applanix corporation in the interface document for POS LV system. The description of position methods whose accuracy is provided as column names in the previous tables is given below in the Table 3:

For the sake of comparison between RTX and RTK in detail, the laps recorded around the University of Wuppertal are considered. The RMS values for the laps are shown in Figure 7a and Figure 7b respectively. In these figures, the location of a peak RMS values is visible across all the laps especially in between samples 10,000 and 15,000. The peak RMS values observed in this interval is due to the presence of tall buildings and underpasses during the course of a lap. The presence of peak RMS values in the areas with tall buildings and underpasses indicate the deterioration of RTK/RTX position updates. In Figure 7b, several smaller RMS peaks are observed followed by minimum values between samples 15,000 and 25,000 indicating that the navigation system is able to re-initialize with minimal position errors after a short duration of RTX signal outage. This ability of RTX position updates to re-initialize the navigation system with minimum errors can also be verified in Figure 6a violin plot as multi-modal distribution of RMS values indicated using two wider sections. In contrast to RTX, the RMS values estimated during RTK position updates in Figure 6a violin plot are uni-modal in nature.

**Figure 8:**
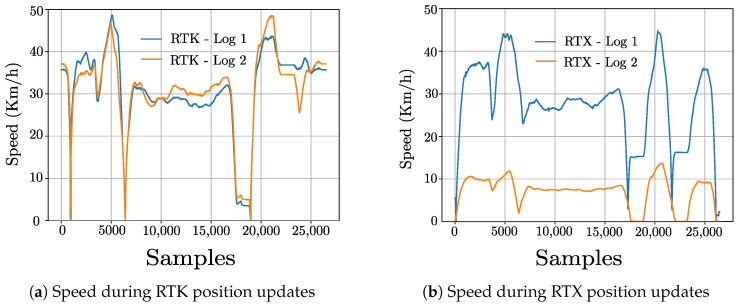
Speed of the vehicle for laps around the University of Wuppertal.

In order to understand the impact of vehicle speed on positioning inaccuracies, the laps recorded aroud the University of Wuppertal are considered again. For this purpose, the vehicle speed for the laps involving RTK and RTX position updates shown in Figure 8a,b are compared against the Mean RMS value plots in Figure 7a and Figure 7b respectively. As general observation for both RTK and RTX updates, the position accuracy is better, i.e., lower Mean RMS values when the vehicle is traveling at low speed or is stationary particularly between samples 15,000 and 20,000. This can be attributed to the fact that GNSS receivers are able to receive position corrections at correct times during lower speeds as compared against higher speeds. As the main objective of the performance evaluation presented in this paper is to analyze various augmentation methods by taking into consideration real-life factors such as the maximum permitted speed in the test region, the dynamic change in traffic situations and various other challenges posed to signal availability, detailed experiments involving different speeds were not performed.

## 6. Conclusions

In this paper, we presented performance tests conducted to compare the position augmentation accuracy of classical DGNSS, RTK and RTX in the cities of Wuppertal and Duesseldorf in Germany. The four test regions are characterized mainly by uneven terrain with tall buildings around the University of Wuppertal, flat terrain with roads of varying width in the city centre of Wuppertal and Duesseldorf and flat terrain in a tunnel section located in the city of Wuppertal. For the performance comparison between the position augmentation techniques, we used horizontal violin plots in order to visualize the distribution of the respective Root Mean Square error values.

As a general observation, the navigation system switched to an available position augmentation method during phases of RTK/RTX signal outages. In particular, in the test region consisting of a tunnel section, the navigation system also switched to a less precise code-based GNSS position augmentation method, minutes after the vehicle entered into this tunnel section. Furthermore, position augmentation services such as RTK and RTX outperformed the classical DGNSS in the test regions with partial to complete open sky conditions. This phenomenon can be observed in the violin plots depicting the distribution of the Root Mean Square values across various position augmentation techniques for each test region.

Also, the Root Mean Square values of DGNSS-based position augmentation are higher than the estimated values with techniques such as RTK and RTX. From the violin plots, the fluctuation of positional accuracy with DGNSS is also observed in an urban environment such as the city centre of Duesseldorf and Wuppertal. Even though the performance of RTK and RTX correction signals deteriorated in a test area consisting of an underpass around the University of Wuppertal, the effectiveness of RTX position updates is seen when the navigation system re-initializes itself after signal outage with minimum position error. As a conclusion from the performance analysis, our results indicate that the RTX augmentation method is proven to be precise and robust even within the urban centres of Duesseldorf and Wuppertal.

In the future, the need for robust position estimation algorithms in autonomous vehicles is inevitable as the results from show that the GNSS position augmentation services such as the classical DGNSS, RTX and RTK are highly affected by the condition of a test region. Therefore, robust position estimation algorithms play an important role especially in areas of complete signal outage using a rough position estimate obtained from the navigation system, a map of the surrounding and scan from an environment perception sensor like an autonomous radar, camera or lidar.

## Figures and Tables

**Table 1:** Percentage ratio of data samples and the corresponding expected accuracy during the laps with RTK position augmentation through the Burgholz tunnel.

Laps	75 m	1 m	0.25 m	0.02 m
1	86.22	8.79	4.98	0.00
2	22.27	69.85	7.86	0.00
3	30.31	18.87	16.19	34.63
4	27.08	61.93	10.98	0.00

**Table 2:** Percentage ratio of data samples and the corresponding expected accuracy during the laps with RTX position augmentation through the Burgholz tunnel.

Laps	75 m	1 m	0.05 m
1	82.20	15.54	2.28
2	53.51	44.26	2.23
3	55.01	42.84	2.15
4	41.75	48.22	10.03

**Table 3:** The Description of position methods whose expected accuracy is provided as column names in Table 1 and Table 2.

Description	Expected Accuracy
C/A mode	75 m
3-dimension DGNSS mode	1 m
Float RTK mode	0.25 m
Integer narrow lane RTK mode	0.02 m
Trimble RTX Mode	0.05 m

## Data Availability

Restrictions apply to the availability of these data. Data was obtained from Aptiv Services Deutschland GmbH and are not available publicly.
